# Cross-Talk between Cancer Cells and the Tumour Microenvironment: The Role of the 5-Lipoxygenase Pathway

**DOI:** 10.3390/ijms18020236

**Published:** 2017-01-24

**Authors:** Gillian Y. Moore, Graham P. Pidgeon

**Affiliations:** Department of Surgery, Trinity College Dublin, Dublin 8, Ireland; mooregy@tcd.ie

**Keywords:** 5-lipoxygenase, leukotrienes, tumour microenvironment, angiogenesis, immune cells, adipose tissue

## Abstract

5-lipoxygenase is an enzyme responsible for the synthesis of a range of bioactive lipids signalling molecules known collectively as eicosanoids. 5-lipoxygenase metabolites such as 5-hydroxyeicosatetraenoic acid (5-HETE) and a number of leukotrienes are mostly derived from arachidonic acid and have been shown to be lipid mediators of inflammation in different pathological states including cancer. Upregulated 5-lipoxygenase expression and metabolite production is found in a number of cancer types and has been shown to be associated with increased tumorigenesis. 5-lipoxygenase activity is present in a number of diverse cell types of the immune system and connective tissue. In this review, we discuss potential routes through which cancer cells may utilise the 5-lipoxygenase pathway to interact with the tumour microenvironment during the development and progression of a tumour. Furthermore, immune-derived 5-lipoxygenase signalling can drive both pro- and anti-tumour effects depending on the immune cell subtype and an overview of evidence for these opposing effects is presented.

## 1. Introduction

The microenvironment of a developing tumour is composed of a dynamic variety of non-cancerous immune and stromal cells within a scaffold of extracellular matrix that a tumour depends upon for sustained growth, invasion and metastasis. Traditionally, anti-cancer drug development has focused on targeting the tumour cell cycle. However, in recent times, there has been a shift in research emphasis from efforts to target cancer cells directly to the attractive alternate or synergistic targeting of components within the tumour microenvironment (TME) [1,2]. In contrast to cancer cells, immune and stromal cells do not undergo rapid and frequent genetic mutations. Therefore, it is hypothesised that targeting the TME should lead to reduced risk of drug resistance and tumour recurrence that is often seen with conventional cancer cell targeted treatments [3]. Targeting the TME is a complex challenge however because of the somewhat ambiguous capacity to both aid and hinder tumorigenesis. The identification of molecular pathways involved in tilting the equilibrium towards a pro-tumorigenic interaction between the TME and tumour cells is key to disrupting immune-stromal cell involvement in cancer growth and progression.

**Figure 1:**
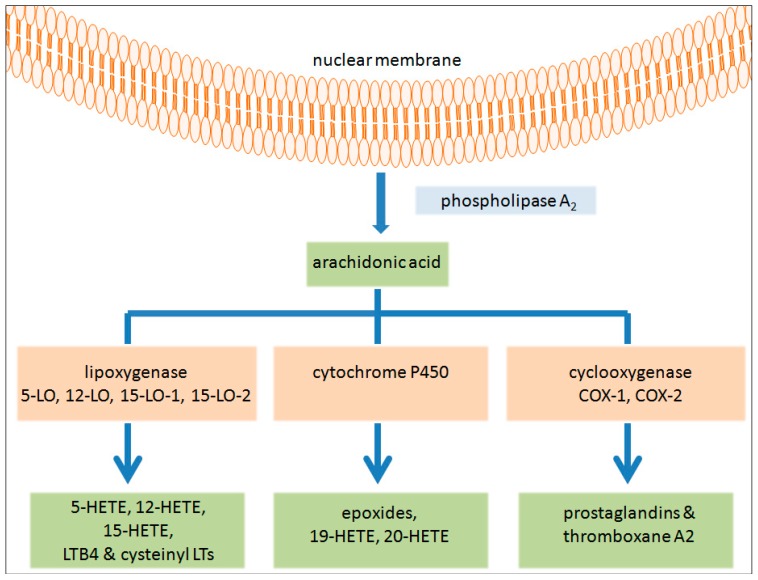
Arachidonic acid metabolism produces bioactive lipid signalling molecules known as eicosanoids: Upon cellular stimulation, phospholipase A2 induces the release of free fatty acids (FFAs), mostly arachidonic acid (AA), from the lipid bilayer of the nucleus. The released AA is metabolised by one of three different enzymatic pathways into the bioactive lipid signalling molecules, known as eicosanoids. 5-lipoxygenase (LO) metabolises AA to 5-HETE, LTB4 and the cysteinyl leukotrienes (LTC4, LTD4 and LTE4), 12-LO metabolises AA to 12-HETE. Both isoforms of 15-LO metabolise AA to 15-HETE and to a lesser extent 12-HETE. Cytochrome P450 metabolises AA to 19-/20-HETE but mostly epoxides. Cyclooxygenase (COX)-1 and 2 metabolises AA to thromboxane A2 or a series of prostaglandins (PGD2, PGE2, PGF2, PGH2 and PGI2).

Eicosanoids are bioactive lipid signalling molecules synthesised mainly from arachidonic acid (AA) through three principle pathways: cyclooxygenase (COX), lipoxygenase (LO) and cytochrome P450 [4] (Figure 1) These eicosanoids have been shown to be involved in numerous pathologic states, including inflammation and cancer [5,6,7,8]. Members of the LO family are classified based on the position of a hydroperoxy group in their metabolite products and include 3-, 5-, 8-, platelet-type 12(*S*)-, epidermal-type 12(*R*)- and 15-LO. In humans, 15-LO consists of two isoforms 15-LO-1 (gene name: *ALOX15*) and 15-LO-2 (*ALOX15B*) [9]. LOs, which are conceivably the least well studied of the three families of arachidonic acid metabolising enzymes, have been indicated to have a role in cancer, both clinically and experimentally in a number of different cancer types. It is a complex relationship however, with both tumour suppressing and tumour promoting activities having been reported for various isoenzymes [10]. 15-LO-1 has been implicated as a tumour promoter of prostate cancer [11,12]. In contrast, 15-LO-1 suppressed chronic myeloid leukaemia and colorectal cancer [13,14,15,16] suggesting a protective role. In the case of breast cancer, data have been somewhat conflicting with both pro- and anti-tumorigenic roles reported [17,18]. The majority of research has reported an anti-cancer role of 15-LO-2 [19,20].

Platelet-type 12-LO and 5-LO can be induced by pro-inflammatory stimuli and are often constituently overexpressed in cancer. Overexpression of 12-LO and an association with disease has been reported in prostate cancer [21,22] and breast cancer [23,24]. Similar results were obtained in a study examining 12-LO mRNA levels between breast cancer tissue and matched normal tissue, and additionally 12-LO levels were found to correlate with tumour staging [24]. Similar to 12-LO, 5-LO is reportedly overexpressed in a number of cancer tissues including prostate, renal, breast, colorectal and pancreatic tumours compared to normal tissues [25,26,27,28,29]. A number of pharmacological inhibitory studies have also indicated a regulatory role of 5-LO in cancer cell proliferation and growth [25,30,31]. In addition to studies that focus on the role of LOs in cancer cells, several studies have implicated LOs, as regulators of the TME [32,33]. For example, it has recently been demonstrated that immune stromal expression of 5-LO and other distal enzymes involved in the synthesis of 5-LO-derived leukotrienes were increased in human oesophageal adenocarcinoma compared to normal oesophagus, suggesting a role of the 5-LO signalling specifically in the TME during tumour development and progression [34].

Given that both cancer cells and non-cancer stromal cells are potent producers of eicosanoids, tumour progression will likely involve an integrated response to eicosanoid signalling pathways. Apart from malignant cells, the TME consists of cells of the immune system, the tumour vasculature and lymphatics, and the extracellular matrix (for a concise description and summary of these cell types, please refer to [35]). This review will focus on the role of 5-LO and its metabolites not only in cancer cells but also cellular components of the TME including macrophages/monocytes, neutrophils, mast cells, B- and T-lymphocytes, and connective tissue cells such as endothelial cells, adipocytes and fibroblasts. Examples from the literature will be provided where 5-LO signalling is involved in the cross-talk between these different cell types. The potential to therapeutically exploit cellular components of the TME through the targeting of this enzymatic pathway will also be discussed. We propose that manipulating LO-dependent AA metabolism in the TME could offer new strategies to block cancer-related inflammation and immune escape.

## 2. 5-Lipoxygenase Pathway in Cancer

The pronounced pro-inflammatory role of metabolites of the 5-LO pathway has been identified in a number of different pathological states including atherosclerosis, Alzheimer’s disease, type 2 diabetes and cancer [5,6,36,37]. The potential of blocking this pathway in the treatment of conditions of chronic inflammation is highlighted by the use of zileuton to improve pulmonary function in children and adults with mild-to-severe asthma [38,39]. Cysteinyl leukotriene receptor 1 and 2 (i.e., CysLT-R1 and CysLT-R2) antagonists such as montelukast and zafirlukast [40,41] are also available in some countries, however zileuton is the only Food and Drug Administration (FDA) approved anti-leukotriene inhibitor.

**Figure 2:**
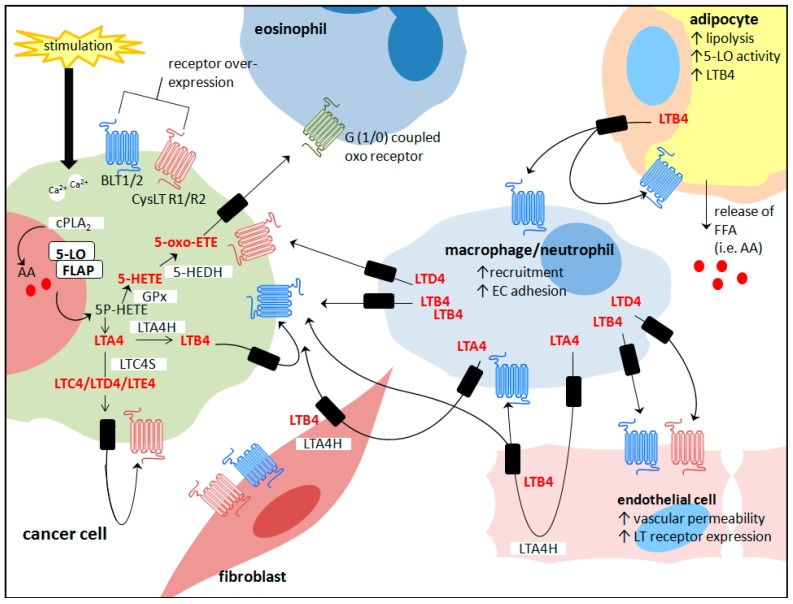
Summary of cross-talk between cancer cells and the tumour microenvironment mediated through the 5-lipoxygenase pathway.

The location of 5-LO in resting cells is cell-type specific, residing in either the cytosol or the euchromatin region of the nucleus [42,43]. Upon p38-dependent-MAPKAP kinase 2-mediated phosphorylation (Ser-271) and activation, 5-LO is translocated to the nuclear membrane [44,45]. Here a vital interaction with FLAP (5-LO activating protein) allows the metabolism of AA, released from the lipid bilayer by Phospholipase A2 [46,47]. AA is transformed to 5-hydroperoxyeicosatetraenoic acid (5-HPETE), which is reduced to 5-hydroxyeicosatetraenoic acid (5-HETE) by ubiquitous Glutathione peroxidase (GPx). 5-HETE can be further converted by 5-LO to LTA4 [48] or oxidised by the microsomal enzyme 5-HEDH to 5-oxo-ETE, a potent chemoattractant of eosinophils [49,50] (Figure 2). Subsequently, LTA4 is oxidised by the enzyme LTA4 hydrolase to LTB4, which is a potent chemoattractant for monocytes, neutrophils and eosinophils, stimulating adhesion and extravasation through vascular endothelial barriers, migration to sites of inflammation, and then their subsequent activation [8]. Alternatively, LTA4 can be converted to LTC4 via the conjugation of glutathione by LTC4 synthase [51]. Furthermore, LTC4 is converted to LTD4 by gamma-glutamyl transferase and subsequently LTD4 is converted to LTE4 by membrane-bound dipeptidase [52]. LTC4, LTD4 and LTE4 are known collectively as the cysteinyl leukotrienes and are reported to have a role in inflammatory cell recruitment, smooth muscle contraction, vessel dilation and permeability [53] (Figure 2). 5-LO is also involved in the synthesis of another type of AA-derived metabolite called Lipoxins, such as LXA4 and LXB4 which have anti-inflammatory and pro-resolving properties [54,55].

The involvement of the 5-LO pathway in the development and progression of tumours has led to its investigation as a potential biomarker in many cancers including oesophageal cancer [34,56]. In a small study of pair-matched benign and malignant prostate tissues (*n* = 22), 5-LO gene and protein expression and 5-HETE production were significantly higher in malignant compared to benign tissue [57]. In another study 5-LO metabolite LTD4 receptor expression was shown to be higher in prostate cancer tissue compared to benign or normal prostate tissue and expression correlated with tumour grade, indicating a role of 5-LO leukotrienes and receptors in prostate cancer progression [26]. In a study of colon adenomas (*n* = 111), 5-LO expression was found to correlate with typical high risk factors for malignant transformation to colorectal adenocarcinoma, suggesting its usefulness as a biomarker in early detection of this cancer type [58]. Additionally, in a small study of 55 sporadic colorectal adenocarcinomas, a positive correlation of 5-LO was found with tumour stage and lymph node metastasis [29].

As mentioned previously, FLAP is a protein necessary for the activity and hence tumorigenicity of 5-LO by transferring AA to 5-LO. In a cohort of 154 breast samples Jiang et al. showed that FLAP mRNA was aberrantly expressed in breast cancer compared to normal tissues and that expression was associated with prognosis [27]. In another study, 5-LO and its metabolite LTB4 were found by immunohistochemistry to be significantly upregulated in pancreatic tumour compared to normal pancreatic ducts [28]. 5-LO expression has also been shown to be expressed during early neoplastic changes in the development of pancreatic cancer, suggesting a role as an early biomarker of disease or as a drug target [59]. The expression of receptors for 5-LO metabolites, including the LTB4 receptors BLT1 and BLT2, have also been shown to be upregulated in a variety of cancers indicating the relevance of the metabolites and accompanying receptors as molecular therapeutic targets [28,34].

There are four identified receptors involved in the 5-LO signalling pathway: The LTB4 receptors 1 and 2 (BLT1 and BLT2) and the LTC4, LTD4 and LTE4 receptors (CysLT-R1 and CysLT-R2) (Figure 2). BLT1 is a high affinity receptor for LTB4 expressed primarily by leukocytes whereas BLT2 is a low affinity receptor for both LTB4 and 12-HETE expressed more ubiquitously [60]. CystLT receptors have been reported in T-lymphocytes, eosinophils, mononuclear cells, macrophages and in certain cases neutrophils [61,62]. Recent studies have indicated a role of these leukotriene receptors in a number of malignancies including urinary bladder, pancreatic, colon and breast cancer [28,63,64,65]. LTB4 receptors BLT1 and BLT2 and CysLT-R1 protein were found to be overexpressed in gastric cancer patient tissue compared to tumour-free normal mucosa while interestingly the 5-LO enzyme protein levels did not vary between the two cohorts [66]. There is far less known about BLT2 in immunoregulation compared to BLT1, however there is increasing evidence to suggest a prominent role of these receptors in a number of different aspects of cancer development, such as proliferation, survival, angiogenesis and Ras-induced transformation and metastasis, further indicating the relevance of 5-LO metabolites and accompanying receptors as molecular therapeutic targets [67,68,69,70].

## 3. Blockade of Leukotriene Synthesis in Cancer Models

While research is still limited in this area, there have been efforts to elucidate the role that 5-LO and the leukotrienes play in cancer and the significance of their upregulation across a number of tumour types. Prostate cancer cells have been shown to constitutively produce 5-HETE in serum free/unstimulated conditions in vitro [71]. Treatment with MK886 (a FLAP inhibitor) and subsequent inhibition of 5-LO signalling led to massive apoptosis of prostate cancer cells as determined by the formation of nucleosomes using Cell Death Detection ELISAs [71]. Subsequently, addition of 5-HETE protected the cells from the effects of MK886 showing that 5-LO activity was crucial to the survival of these cells. In vivo, 5-HETE in metastatic prostate tumours in mice xenografts was significantly higher than that of in situ tumours while treatment with zileuton resulted in fewer tumour masses [72]. In the same study, Meng et al. also showed that treatment of human prostate cancer cells with zileuton decreased their migratory ability in trans-well assays. Efforts to characterise the observed regulatory role of 5-LO in prostate cancer are growing. For example, the induction of apoptosis by 5-LO inhibition in prostate cancer cells was reported to occur via a downregulation of protein kinase C and this apoptotic effect could be blocked by the addition of exogenous 5-HETE [73]. Most recently, 5-LO inhibition in prostate cancer in vitro has been shown to suppress the protein level and activity of oncogenic c-Myc, suggesting a novel mode of 5-LO action [74]. New evidence has indicated a reduction in the stemness factors Nanog and c-Myc in prostate cancer stem cells indicating that 5-LO may play a role in maintaining stemness in prostate cancer [75]. Thus, effective drug targeting of 5-LO in prostate cancer may stop tumour development and growth and reduce recurrence. 5-LO inhibitors were shown to have anti-proliferative effects in renal cell carcinoma cells in a dose and time-dependant manner [76]. Several pancreatic cancer cell lines have also been shown to express 5-LO at both the gene and protein level, with little or no expression determined in normal pancreatic ductal cells [28]. Inhibition of 5-LO with REV 5901 or FLAP with MK886 led to reduced pancreatic cell growth which could be reversed by the addition of 5-HETE, indicating a critical growth stimulatory role of 5-LO and its metabolite [30]. This pro-proliferative effect of 5-HETE, at least in pancreatic cancer cells, has been shown to occur via activation of the PI3 kinase/Akt pathway [77]. A number LO inhibitors including the specific 5-LO inhibitor REV5901 altered pancreatic cancer cell proliferation through an induction of apoptosis with decreased Bcl2 and Mcl2 and increased Bax expression being reported [78]. Furthermore Tong et al. documented similar results in an in vivo athymic mouse xenograft model of pancreatic cancer where 5-LO inhibition was also shown to decrease proliferation and cause apoptosis in the tumour mass [78]. A report has demonstrated that 5-LO inhibitors can elicit cytotoxic and anti-proliferative effects on tumour cells, independently of 5-LO enzyme suppression and thus careful consideration must be taken when interpreting these pharmacological inhibitor data [79].

The 5-LO pathway is not just relevant to solid malignancies as there has been recent evidence demonstrating the utility of 5-LO inhibition as a clinical approach for cancer-stem-cell driven acute myeloid leukemia [80]. Genetic or pharmacological inhibition of 5-LO in disease models, rendered the enzyme inactive and had the knock-on effect of suppressing Wnt signalling, a process critical for maintenance of the cancer stem cell population. Furthermore a murine model of BCR/ABL induced CML failed to manifest in the absence of 5-LO, while pharmacological inhibition of 5-LO function in combination with BCR-ABL kinase inhibitor imatinib significantly prolonged survival of CML mice [81]. Aside from this emerging role in haematological malignancies, 5-LO signalling may have important interactions with non-cancerous white blood cells in the vicinity of a tumour and we explore this research area in the following section.

## 4. 5-Lipoxygenase and Immune Regulation

### 4.1. 5-Lipoxygenase Signalling and Infiltrating Myeloid Derived Cells

It is well established that dysplastic and malignant tissues are infiltrated by numerous types of leukocytes, but the specific role of these individual leukocytes in the cancer setting remains somewhat elusive [82]. Although the main function of the immune system is immune surveillance and protection from pathogens, including the prevention of tumour onset, there is contradictory experimental and clinical evidence demonstrating that tumours arise and thrive at sites of inflammation [83,84]. The majority of studies in the literature have focused on 5-LO and its metabolites in epithelial cells alone and they have indicated a role in regulating tumorigenesis [72,76,85,86]. However there is mounting evidence to suggest that 5-LO plays a key role in the recruitment and expansion of hematopoietic immune cells and that stromal cells are the main source of 5-LO metabolite products that can potentially drive tumorigenesis [87]. 5-LO is expressed in a number of leukocytes including neutrophils, monocytes, macrophages, mast cells, basophils, eosinophils and B-cells and under certain stimuli they can synthesise leukotrienes that can induce inflammation by attracting and activating more leukocytes [88]. In recent times, there have been efforts to identify the mechanisms through which LTB4 acts as a chemoattractant. It has been reported that LTB4 and LTB4 synthesising enzymes are packaged into neutrophil-released exosomes [89]. These LTB4 containing exosomes can form a gradient and elicit autocrine and paracrine stimulation of a neutrophil and other following cells towards a primary chemoattractant. Neutrophils and macrophages are the main producers of LTB4 while macrophages, eosinophils, basophils and mast cells are all shown to secrete varying levels of the CysLTs [90].

Associated inflammation in the TME induces tumour development by providing fuels to nurture epithelial cell growth and pro-angiogenic factors for vascularisation and angiogenesis. For instance, increased levels of LTB4 were associated with the chronically inflamed pleura in the lung of tuberculosis or cancer patients [91]. Pleural macrophages resident in the lung were found to release significant amounts of LTB4 earlier and stronger than resident mesothelial cells during the initiation of pleural inflammation and neutrophil recruitment, which are activated to release more LTB4 [91]. In concordance with this finding, blockage of 5-LO expression was shown to deplete neutrophil numbers infiltrating dysplastic lesions of the colon in a mouse model of polyposis [92]. The exact role of macrophages in tumorigenesis is controversial with conflicting evidence suggesting that macrophage tumour infiltration improves patient prognosis in some cancers yet decreases it in others [93]. Interestingly, a 5-LO knockout murine model of colon polyposis demonstrated a reduced polyp burden and increased tumour infiltrating macrophages and suggesting a pro-tumorigenic role of 5-LO in polyp formation but a seemingly protective effect of infiltrating macrophages in this process [92]. It was hypothesised that suppression of 5-LO may result in a previously observed increase in IL-12, a potent cytokine known to stimulate macrophages with an anti-tumour phenotype [94].

Tumour-associated macrophages (TAMs) are found in most cancers and are usually pro-tumorigenic [95]. They have been shown to have M2-type phenotype, secreting a vast array of cytokines, chemokines, growth factors and proteolytic enzymes. They have been described as obligate partners of tumour cell migration, invasion and metastasis, immune suppression and tumour angiogenesis [96]. While they can be found in well vascularised areas, hypoxic tumour regions where the TAMs can interact closely with the tumour cells tend to be an abundant site of TAMs due to a release of chemoattractants such as VEGF and MCP-1 [97]. A recent study found that 5-LO expression in ovarian tumour tissue was strongly associated with the density of TAMs in hypoxic areas, suggesting a role in TAM homing [31]. In vitro, hypoxia was shown to induce higher 5-LO expression and metabolite production in ovarian cancer cell line models. Increased 5-LO metabolite production was shown to induce migration and invasion of macrophages in vitro, through an upregulation of MMP7. Developing their hypothesis further, Wen et al. demonstrated that blocking 5-LO activity by zileuton in mouse ovarian tumour xenografts decreased 5-LO metabolite-induced TAM recruitment to hypoxic areas and TAM MMP-7 expression. Tumour growth was suppressed and considering 5-LO activity has been shown to be involved in other processes including tumour proliferation and tumour angiogenesis, it likely that multiple pathways are being affected by 5-LO inhibition in this xenograft model and not just immune cell infiltration [31].

Inflammation is crucial to the resolution of infection and injury, however there must be a tight balance between pro- and anti-inflammatory signals to prevent untoward and excessive damage to host cells and tissues [98,99]. Aberrant levels of 5-LO inflammatory leukotrienes have been extensively implicated in the pathogenesis of a variety of respiratory diseases including lung malignancies [100,101,102]. Alveolar macrophages which constitute over 90 per cent of the cellular component of bronchial alveolar lavages (BALs) are a major source of LTB4 [103]. This LTB4 drives recruitment of leukocytes and subsequent adhesion to vasculature, stimulating the production of pro-inflammatory cytokines from other macrophages and lymphocytes. 5-LO metabolite levels have been shown to be significantly higher in BALs of smokers than ex-smokers [103]. Evidence suggests that tobacco negatively impacts regulatory mechanisms in place to maintain balanced 5-LO activity and LTB4 production in the lung, by abrogating its inactivation and oxidation to LTB4OH, both constituently and in response to inflammatory stimuli [104]. It is now hypothesised that these sustained high levels of LTB4 in the lung microenvironment may contribute to tobacco exacerbated respiratory diseases including lung cancer.

As previously discussed, cysteinyl LT receptors are upregulated in colon cancer patients [64,105]. Macrophage and leukocyte derived LTD4 present in the TME has been shown to contribute to chronic inflammation and to increase proliferation and migration of colon cancer cells [106]. It is known that established colon cancer cell lines express 5-LO and produce LTB4 in vitro [86]. A murine study focusing on hematopoietic derived 5-LO, observed that specific deletion of this enzyme from bone marrow decreases polyp formation despite the presence of 5-LO proficient epithelial cells in these mice, indicating for the first time the importance of immune cell contribution to 5-LO activity [87]. Mast cells not only produce pro-inflammatory eicosanoid metabolites but are also regulated by these signalling molecules in both a paracrine and autocrine fashion [107]. Mast cells, which have previously been shown to be critical for the formation of pre-neoplastic colon polyps [108], were the most significantly reduced infiltrating myeloid cell type. Isolated mast cells from this population were unable to produce 5-LO metabolites and lost their capacity to stimulate intestinal epithelial proliferation and recruitment of myeloid derived suppressor cells (MDSCs), a particular subset of immune cells capable of inhibiting protective anti-tumour cytotoxic T-cells. They also lost their ability to modulate the immunosuppressive activity of arginase in MDSCs [87,109]. One recent study demonstrated an increased focal production of 5-LO LTB4 in human colon polyps compared to normal healthy adjacent tissues [110]. Immune cells of the TME in addition to dysplastic epithelial cells are likely contributors to these increased metabolite levels. In this same study increased serum levels of LTB4 and other pro-inflammatory cytokines were observed in a mouse model of polyposis. It is well known that tumour-associated inflammation is a prerequisite for the tissue changes required for early dysplastic lesion formation. This process involves infiltrating myeloid cells such as mast cells, macrophages, MDSCs and their associated pro-inflammatory cytokines such as TNF-α, IL-1β, IL-6 and VEGF. Zileuton treated polyps were found to have significantly lower levels of infiltrating macrophages, mast cells, MDSCs (>50%–60% lower) which Gounaris et al. suggest is responsible for the lower incidence of polyps in these mice. In another study, pancreatic ductal adenocarcinoma lesions were significantly less frequent in 5-LO knockout mice and a decrease of infiltrating pancreatic mast cells was also noted [111]. This was further supported in human pancreatic ductal adenocarcinoma where increased 5-LO immunostaining was associated with increased mast cell numbers [111]. Therefore targeting myeloid 5-LO signalling may be an effective therapeutic strategy for polyposis and potentially colorectal and pancreatic cancer [110,111].

Similar to smoking, excessive alcohol is a risk factor for cancer development, particularly in oral cancer [112,113]. A study has demonstrated a relationship between ethanol related oral carcinoma and eicosanoids. 5-LO and COX-2 expression were found to be enhanced in dysplastic areas and squamous cell carcinoma of the tongue in a mouse model of ethanol promoted 4-Nitroquinoline 1-oxide (4NQO)-induced oral carcinogenesis compared to the chemically-induced tumour formation alone [114]. Interestingly these promoting effects of ethanol were suppressed in ALOX5−/− mice with the incidence of lesions being significantly less than in mice with functioning 5-LO, in addition to reduced cell proliferation, angiogenesis and inflammation in the tongue. Reduced numbers of mast cells in the TME was demonstrated to be responsible, at least in part for this significantly dampened tumour-associated inflammation [114]. The observed decrease in cell proliferation may be due to a reduced stimulatory interaction between the tumour epithelium and the mast cells. They concluded from this work that ethanol can induce a faster malignant transformation in the model at least in part through the activation of the 5-LO pathway.

### 4.2. 5-Lipoxygenase Signalling and Lymphocytes

5-LO has been shown to play a role in the adaptive immune system. 5-LO is expressed in mantle zone B-cells and plays an important role in regulating follicular B-cell fate and generating follicular B helper T-cells (Tfh) and memory B-cells required to mount an effective humoural response and to protect the host [115]. Genetic inhibition of bone marrow stem cell-derived 5-LO exacerbates a mouse model of enteric colitis demonstrating the critical role of 5-LO in B-cell humoural response to pathogens [115]. Interestingly, in this same study, the cellular growth of mantle cell lymphoma (a tumour composed of primary B-cells) also seems to depend, at least in part on 5-LO expression and activity, although the nature of this relationship in this unique tumour has not yet been studied.

Tumours are able to resist immune attack either by manipulating host-immune system suppressive pathways or by immune exclusion or ignorance [82]. In the pathological state of cancer, primary B-cell lymphocytes may act as a modulator of abnormal adaptive immune responses. B-cell infiltration in the TME is associated with improved prognosis for some cancer types [116,117]; however, more recent data support the role of a newly described immunosuppressive sub-population of IL-10 producing regulatory B-cells (tBregs) [118]. B-cells are known to produce LTB4 and to express the receptor BLT1 and BLT2 [119]. CysLT production has not been described for these cells. In breast cancer, these tumour evoked tBregs were found to induce expansion of pro-tumorigenic regulatory T-cells, increase tumour burden and also appear to stimulate metastasis to the lung [32]. 5-LO metabolites are known to be potent inducers of tumour-associated inflammation, however a relatively new role in cancer immune evasion is now emerging. Production of 5-LO metabolites, namely LTB4 by non-metastatic breast cancer cells was shown to induce the activation of immunosuppressive tBregs via PPARα, promoting cancer escape and metastasis in a mouse model [33]. This effect was almost entirely lost when mice were engrafted with ex vivo MK886-pretreated tBregs, indicating the importance of 5-LO activity not only in the cancer cells but in the B-cells also. Pharmacological inhibitor studies indicated that the tBreg 5-LO/LT/PPARα activity was required for the generation of pro-metastatic FOXP3+ Treg cells and the subsequent suppression of cytotoxic CD8+ T-cells. Notably, Wejksza et al. demonstrated that induction of tBregs required the activation of 5-LO in the B-cells, not just the cancer cells, as specific inhibitors of the 5-LO pathway completely abrogated this process. This is the first defined example reported in the literature on tBreg activity in the cancer setting. Wejksza et al. suggest B-cell 5-LO activity is needed to enhance the stimulatory effect of the cancer cell derived leukotrienes and/or to promote survival of cancer cells, via upregulation of key factors, such as VEGF [33].

There are numerous T-cell populations within the TME that infiltrate tumour margins and within draining lymph nodes, the spleen and the thymus. Recently, purified T-cells have been reported to express 5-LO at both the gene and protein level and to be capable of LTB4 and CysLT synthesis when supplied with an exogenous source of AA [120]. However there has been conflicting evidence regarding T-cell expression of 5-LO over the past two decades with supporting and negative data published [120]. Thus, further independent studies are needed to clarify these findings. T-cells have been reported to express BLT1 and BLT2 receptors [90] and the CystLT receptors CystLT-R1 and CystLT-R2 [62]. It has been reported that the metabolites LTB4 and CysLTs are potent chemoattractants for T-cells [121,122]. T-cell derived 5-LO signalling has opposing anti- and pro-tumorigenic roles. In a murine model of cervical cancer BLT1 expression was necessary for the recruitment and anti-tumour response of CD8+ T-cells [123]. In another cancer setting, injected murine lung cancer cells were found to propagate larger tumours when injected into 5-LO knockout mice compared to wild type mice [124]. Poczobutt et al. determined that 5-LO activity in the tumour microenvironment has an anti-tumorigenic role. CD4+ and CD8+ T-cells, and NK cells were decreased in tumour bearing lungs of 5-LO knockout mice compared to wild-type while myeloid cell frequencies remained unchanged. Neutralisation of CD8+ T-cells in wild-type mice resulted in similar sized tumours to the 5-LO knockdown mice, suggesting that the 5-LO pathway protects against cancer progression via a mechanism dependent on CD8+ T-cells.

In contrast to these two studies, other evidence exists to suggest an alternate pro-tumorigenic role of the 5-LO pathway in T-cells, for example in the process of Treg modulation. CD4+FOXP3+ T-cells are regulatory T-cells involved in suppressing the activities of T helper cells and regulating appropriate immune responses to infection. In the cancer setting there is evidence supporting both anti-tumour roles, suppressing or delaying inflammation mediated tumour development and a contradictory pro-inflammatory and pro-tumour role promoting tumour immune escape [84]. One explanation for these apparently opposing roles is the ability of Tregs to convert into IL-17 producing TH17 cells under certain stimuli [125]. This subset of CD4+FOXP3+ cells express RORyt and are known to mediate pathogenic pro-tumorigenic effects in human tumours such as colorectal cancer [125]. It is interesting to note that in the mouse model of polyposis these pro-inflammatory CD4+FOXP3+RORyt+ cells were significantly decreased in the spleen and mesenteric lymph nodes following zileuton treatment, suggesting an involvement of 5-LO signalling in the regulation of these cells [125].

**Figure 3:**
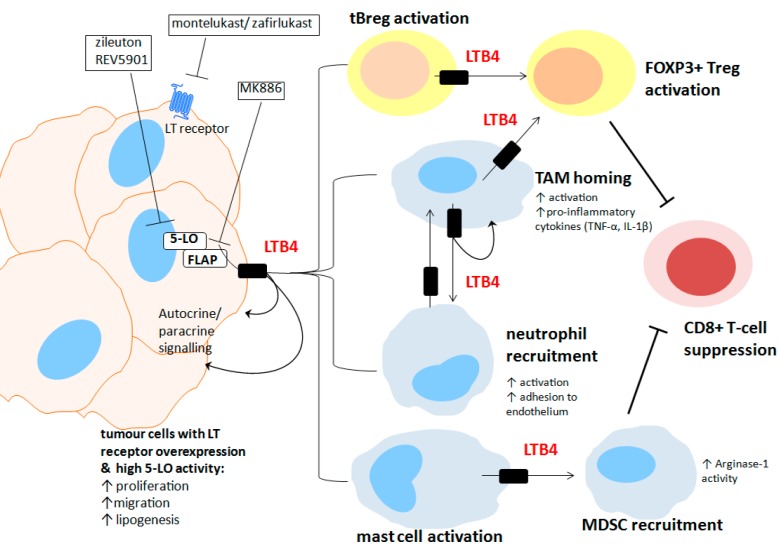
Pro-tumorigenic interactions between cancer cells and immune cells in the tumour microenvironment are mediated by the 5-lipoxygenase signalling pathway: Cancer cell produced LTB4 can stimulate increased proliferation, migration and lipogenesis (i.e., free fatty acid release) in an autocrine fashion and can also stimulate these processes in other tumour cells in the tumour mass. Cancer cell LTB4 paracrine signalling can induce and activate a number of immune cell types in the TME including tumour-associated macrophages (TAMs), neutrophils and mast cells. This results in increased production of pro-inflammatory signalling molecules (e.g., TNF-α, and IL-1β), increased vasculature adhesion and homing to the TME with further activation of other leukocytes. Cancer-associated stimulation of regulatory B-cell (tBreg) LTB4 production leads to activation of regulatory FOXP3+ Tregs which suppress anti-tumorigenic cytotoxic CD8+ T-cells. Mast cell activation through 5-LO signalling results in an increase in their LTB4 activity which can stimulate myeloid derived suppressor cell (MDSC) recruitment and an increase in MDSC arginase-1 activity that can subsequently suppress cytotoxic CD8+ T-cell activity.

In summary myeloid and lymphoid derived immune cells such as macrophages, mast cells, neutrophils, B-cells and various T-cell subtypes, in addition to dysplastic or cancerous cells, are likely contributors to the increased 5-LO metabolite levels in the TME. Figure 3 summarises the 5-LO signalling interactions between cancer cells and immune cell types that have been implicated in enhanced tumorigenicity. Given the complexity of the 5-LO pathway and the opposing roles evidenced in the literature for certain immune cell subtypes, caution should be used in targeting this pathway in cancer.

## 5. 5-Lipoxygenase and Tumour Angiogenesis

### 5.1. Cancer Cell Derived 5-Lipoxygenase in Angiogenesis

There is some evidence for the expression of 5-LO in endothelial cells in the literature under different pathological conditions. Human cytomegalovirus (HCMV)-infected placental tissue was shown to have endothelial cell 5-LO overexpression while in vitro HCMV-infection of human umbilical vein endothelial cells (HUVECs) stimulated 5-LO expression [126]. 5-LO has also been found to be overexpressed in the associated endothelial cells in pulmonary hypertension [127,128]. In a recent publication, it was found that 5-LO expression and LTB4 levels were elevated in Kaposi sarcoma (KS) lesions, latent endothelial cells and de novo endothelial cells [129]. KS is a relatively rare and highly angiogenic tumour of proliferative endothelial cells caused by the KS-associated herpes virus (KSHV) that tends to develop in immunocompromised individuals, such as HIV-infected patients. In a series of experiments, Sharma-Walia et al. provided evidence to suggest the 5-LO signalling pathway is important in the biology and pathogenesis of KSHV [129]. Pharmacological blocking of 5-LO hindered the expression of a number of immunomodulatory genes used by KSHV to remain undetected by host immune surveillance mechanisms during latency, and also decreased monocyte adhesion and trans-endothelial migration. 5-LO signalling was also found to be linked to lipogenesis, the process of fatty acid synthesis, which has been found to be a critical process in the survival of endothelial cells latently infected with KSHV [130].

In general, endothelial cells do not have the capacity to produce leukotrienes de novo due to low or negligible enzyme expression, however they do express both LTB4 receptors and CysLT receptors [90], suggesting the probability of interactions between leukotriene synthesising cancer cells and non-cancer cells in the TME. BLT1 and BLT2 have been found to be upregulated on the surface of endothelial cells following exposure to the pro-inflammatory cytokines TNF-α, IL-1β and LTB4 itself and this receptor upregulation led to a pro-inflammatory phenotype with the release of monocyte chemotactic protein (MCP)-1 [131]. Further study of the effect of LTB4-induced signalling in endothelial cells has been somewhat hampered by the finding that a number of common LTB4 receptor blockers or antagonists used in the literature have been identified as having intrinsic agonist effects, making it difficult to fully interpret in vitro receptor blockade studies to date [132]. Alternative approaches such as genetic knockdowns of the receptor may provide clearer insight.

Most in vitro studies of tumour angiogenesis depend on primary endothelial cells or endothelial cell lines derived from normal human blood vessels, which by their nature are not truly representative of endothelial cells that have been altered or conditioned by the TME and the aberrant growth factor and chemokine signalling associated with this environment. Endothelial cells derived directly from primary tumour tissue are therefore a more desirable model. An involvement of 5-LO in glioma tumorigenesis has been demonstrated [133]. A recent publication found that glioma derived microvascular cells formed higher numbers of tubule structures in the presence or absence of VEGF over the same time period compared to standard lines used such as the HUVECs and ECV304. Synthetic dl-nordihydroguaiaretic acid (i.e., Nordy), a chiral form of the natural 5-LO inhibitor nordihydroguaiaretic acid present in plants, has been shown to markedly reduce the tubule formation by glioma derived endothelial cells, suggesting that the anti-tumour activity of this compound can be attributed at least in part to its ability to interfere with tumour angiogenesis [134].

The zebrafish developmental model has become a popular tool for pharmacological screening due to the ease of drug administration and high throughput nature of the model [135]. Generation of a transgenic zebrafish Tg (fli:EGFP) which expresses green fluorescent protein in the blood vessels formed along the dorsal side of the embryo has extended the utility of this model to testing novel small molecule anti-angiogenic compounds. Advances in tumour xenotransplantation in the zebrafish model now allow for the investigation of tumour endothelial cell cross-talk in processes such as neovascularisation and metastasis [136]. A recent publication using the specific 5-LO inhibitor Nordy did not affect normal zebrafish embryogenesis or developmental angiogenesis. This is in contrast to other anti-angiogenics such as VEGF inhibitor Sunitinib, which completely obliterated vessel formation in the developing embryo [137]. Only inhibition of neo-angiogenesis induced by xenografted glioma cancer cells was observed, suggesting 5-LO has no involvement in zebrafish developmental angiogenesis [138]. Nordy treatment also blocked the invasion of the xenotransplanted cells to distal regions of the embryo. This study highlights clearly that while developmental in vivo models are invaluable tools in research we need to devise models that best mimic the human TME so that tumour specific responses are not overlooked.

### 5.2. 5-Lipoxygenase Signalling and VEGF-Mediated Angiogenesis

Aberrant lipid signalling is evident in many diseases with vascular pathology including cancer. Although one publication on oxygen-induced retinopathy has indicated an anti-angiogenic role of 5-LO [139], the majority of evidence in a number of different pathological states, particularly cancer, indicates a pro-angiogenic role [138,140,141]. For instance, human malignant mesothelioma cells express catalytically active 5-LO and synthesise 5-HETE and LTA4, but not LTB4. These metabolites were found to upregulate a major pro-angiogenic factor, VEGF, in human malignant mesothelioma in vitro [142]. It can be hypothesised that 5-LO promotes tumour development by a dual mechanism: a direct proliferative stimulus on cancer cells, potentially through VEGF-suppressed apoptosis, and by driving a pro-angiogenic response by endothelial cells in the TME. Indeed Ramano et al., observed that 5-LO products upregulate VEGF transcription in HUVECs [142]. In cigarette smoke-induced inflammation of the colon in a mouse model, 5-LO was found to be upregulated in adenomas. An accompanied increase in MMP9 and VEGF, which could be blocked by pharmacologically inhibiting 5-LO, suggested that 5-LO expression in the inflamed colon may be involved in the angiogenic and inflammatory process of adenoma formation [141]. Furthermore, in a study of 45 colorectal adenomas, 5-LO expression was found to correlate with microvessel density as indicated by CD105, suggesting tumour 5-LO activity may modulate the formation of blood vessels in this cancer [143]. Phytochemicals in dietary black and brown rice bran have been shown to reduce tumour burden and tumour blood vessel formation in a mouse model of colon cancer and recent evidence suggests the mechanism of these natural chemicals is mediated through eicosanoid signalling pathways [144]. Transcript and protein expression of COX-2 and 5-LO as well as the production of their metabolites PGE2 and LTB4 were downregulated in addition to the major angiogenic factor VEGF. While definitive evidence was not provided, they speculate that the repression of VEGF and thus VEGF-induced tumour growth and neoangiogenesis was mediated through a downregulation of these two eicosanoid enzymes, providing another example where 5-LO signalling is potentially involved in angiogenesis [144]. DMBA-induced mammary gland tumours in mouse models were shown to progressively express 5-LO [140]. Treatment with zileuton not only reduced tumour burden but also significantly reduced tumour-associated inflammation (indicated by immune infiltrate) and angiogenesis (indicated by microvessel density), implicating 5-LO as an important player in DMBA-induced mammary tumour development and angiogenesis. Similar to Ye et al., 5-LO blocking by zileuton was shown to decrease VEGF and MMP2 expression, indicating the crucial role of 5-LO in modulating the angiogenic process [140].

### 5.3. Endothelial Cell Transcellular Synthesis of Leukotrienes

Human endothelial cells do not normally express 5-LO and therefore do not have the ability to metabolise AA to the intermediate 5-HETE and LTA4 [90]. Endothelial cells do however possess LTA4 hydrolase activity and therefore can convert the exogenous leukotriene intermediate LTA4, provided from another cellular source in the TME, to LTB4 and the cysteinyl leukotrienes LTC4, LTD4 and LTE4, in a process known as transcellular synthesis [145,146] (Figure 2). Co-cultures of S^35^-cysteine labelled endothelial cells with polymorphonuclear leukocytes (PMNL) have shown clear evidence of transcellular metabolism of PMNL-derived LTA4 to cysteinyl leukotrienes. LTB4 levels were also higher in the co-culture compared to PMNLs alone suggesting the interaction of vascular endothelium with activated leukocytes modulates the quantity and type of leukotrienes synthesised [147]. With multiple donors and acceptors possible (i.e., endothelial cells, erythrocytes and platelets), this likely common event could significantly influence the biosynthetic spectrum of active eicosanoid products synthesised by the TME during cancer development.

LTB4 is a potent stimulator of neutrophil migration and adhesion [148] and indeed the function and movement of other immune subtypes [149,150,151]. In a number studies LTB4 stimulation of endothelial cells in the presence of neutrophils led to increased vascular permeability both in vitro and in vivo [152,153]. Released heparin binding proteins from LTB4-activated neutrophils were subsequently identified as mediators of this observed vascular permeability [154]. LTB4 has also been shown to induce endothelial cell migration, tubule formation and induce VEGF-mediated angiogenesis via BLT2 in vivo [68]. Interactions with circulating neutrophils and subsequent synthesis of LTB4 through this transcellular intermediate sharing mechanism could result in autocrine activation of endothelial cells and increased permeability and the subsequent paracrine stimulation and migration of more neutrophils to the TME. It also raises the question whether endothelial cells in the vicinity of a tumour can receive leukotriene intermediates from tumour epithelial cells to produce LTB4.

The synthesis of CystLTs by endothelial cells through this intermediate sharing with donor PMNLs is associated with the synthesis of CysLT-R2 [155], the induced expression of endothelial adhesion molecules [156,157] and the activation of a pro-inflammatory endothelial cell phenotype [158]. CysLTs have also been shown to induce inflammatory signals and proliferation of endothelial cells through CystLT-R1 and CystLT-R2 [159]. As mentioned previously, there are numerous examples of tumours that express an active form of 5-LO and receptors to these metabolites. Although the studies mentioned here are not cancer specific, they do provide some insight into how immune cells or indeed tumour cells, and endothelial cells may interact through the 5-LO signalling pathways, and how dysfunctionally activated endothelial cells could play a role in pathological angiogenesis, inflammation and subsequent metastasis. A number of tumours are known to overexpress LTA4 hydrolase [160], therefore the transcellular synthesis of leukotrienes using LTA4 produced by leukocytes, particularly neutrophils could also contribute to the overproduction of leukotrienes and ensuing inflammation associated with the TME [4].

### 5.4. 5-Lipoxygenase and Cyclooxygenase-2 Shunting in Tumour Angiogenesis

**Figure 4:**
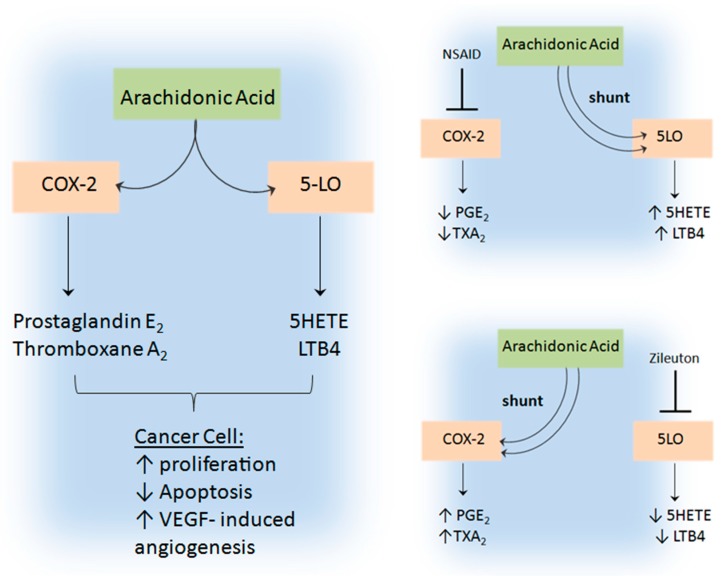
Pharmacological inhibition of the cyclooxygenase-2 signalling pathway results in a shunting of arachidonic acid to the 5-lipoxygenase pathway or vice versa.

As previously discussed, 5-LO is increased in colon cancer specimens and colonic dysplastic lesions/polyps [86]. 5-LO has strong converging roles with another major eicosanoid involved in cancer, cyclooxygenase-2 (COX-2), which has been strongly linked to tumorigenesis and angiogenesis [161]. Given that both enzymes utilise the same substrate of AA and have converging targets of VEGF expression and release, redundancy of these pathways has been suggested [161]. Recent pharmacological inhibition studies have indicated a shunting mechanism between these two arms of the AA pathway where blocking of one enzymatic pathway leads an upregulation of the other [141,162,163] (Figure 4). Ye et al. showed that cigarette smoke can induce adenoma formation in the colon of mice and that inhibition with the 5-LO inhibitor zileuton not only reduced 5-LO expression but also downregulated VEGF, MMP2 and MMP9 [141]. In a subsequent study by this group COX-2 inhibition was shown to result in an upregulation of 5-LO LTB4 [163]. This shunting process was not observed when 5-LO was inhibited, suggesting 5-LO inhibitors are more effective in this mouse xenograft model of cigarette smoked-induced colon cancer. Park et al. demonstrated that inhibition of COX-2 activity in vitro led to an increased LTB4 in head and neck squamous cell carcinoma (HNSCC). Knock-down of COX-2 showed only modest effects to cell proliferation in several cell lines but combined knock-down of both COX-2 and 5-LO had superior inhibitory effects on tumour cell proliferation and VEGF production [162].

Additionally, genetic disruption of 5-LO in a mouse model of ethanol-induced oral carcinoma resulted in activation of COX-2 and an increase in COX-2 metabolites thought to be produced mainly by inflammatory cells [114,161] This evidence suggests dual inhibition of both these pathways may have a superior anti-cancer effect in the suppression of angiogenesis. These findings highlight the question of whether other LO enzymes whose expression are often associated with cancer are also involved in this integrated system.

## 6. 5-Lipoxygenase and Adipose Tissue

In our discussion of the TME, it is important to note the importance of adipose tissue as a compartment of cells that interact with cancer. There is a growing appreciation for the role played by adipocytes and adipose tissue in supporting tumorigenesis and metastasis, and in driving the increased incidence and mortality rates of cancers that grow in the vicinity of adipose tissue such as breast, prostate, endometrial, gastrointestinal and renal cancer [164,165,166,167,168]. In obesity, expanded adipose tissue depots are characterised by increased inflammatory cell infiltration (i.e., macrophages and lymphocytes) and heightened adipokine and cytokine secretions, creating a state of chronic low grade inflammation [165]. Similarities between the adipose tissue-associated microenvironment of metabolic disease/type II diabetes and the TME of adipose tissue-dominated tumours have been highlighted, indicating that the molecular pathways involved may be related [165].

**Figure 5:**
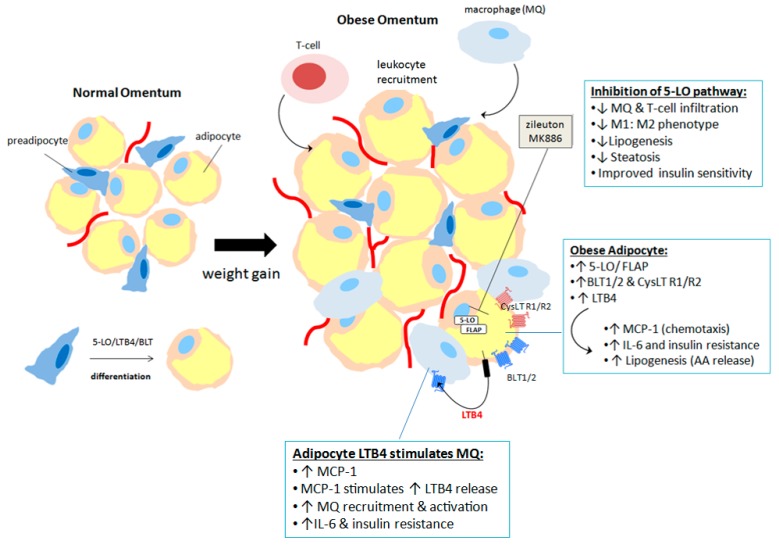
Increased 5-lipoxygenase activity in adipose tissue is associated with obesity: 5-LO signalling has been shown to be involved in preadipocyte differentiation to mature an adipocyte. Adipocytes derived from obese adipose tissue have increased 5-LO/FLAP expression and increased leukotriene receptor expression. Upregulated LTB4 production leads to increased MCP-1 production and increased macrophage (MQ) and T-cell recruitment to the fat. Increased LTB4 signalling also leads to upregulated lipogenesis and thus increased arachidonic acid (AA) availability for further leukotriene synthesis by adipocytes and adipose tissue associated MQs (ATM). LTB4 stimulates MQ production of MCP-1, further LTB4 production, increased pro-inflammatory cytokines such as IL-6 and increased macrophage homing to the fat. Pharmacological blocking of 5-LO signalling in adipose tissue decreases lipogenesis, leukocyte infiltration, decreases M1 type MQs and increases M2 type MQs, overall leading to improved insulin sensitivity and liver function.

Dietary n-6 polyunsaturated fats, such as AA play an important role in the pathogenesis of obesity-related complications in peripheral tissues and organs because they provide fatty acids as raw material for the synthesis of pro-inflammatory eicosanoids [169]. In the past few years 5-LO has emerged as a potential target for obesity-driven disorders such as insulin resistance and metabolic dysfunction [35]. Firstly, all molecular components of the 5-LO pathway required for leukotriene synthesis are reported to be constituently expressed in adipose tissue, in both the adipocyte fraction and the stromal vascular fraction, as well as the two LTB4 receptors (BLT1 and BLT2) and the two CysLT receptors (CysLT-R1 and CysLT-R2) [170] (Figure 5). Secondly, LTB4 signalling has been shown to be critical for the differentiation of preadipocytes to mature adipocytes [171] (Figure 5) and similarly to leptin, MCP-1 and other major adipokines, 5-LO derived leukotrienes have been shown to be elevated in obese adipose tissue [172,173,174,175]. Increased LTB4 concentration has been associated with increased leukocyte chemoattractants and activators such as TNF-α, IL-6 and MCP1 [170] and indeed MCP-1 has been shown to stimulate LTB4 in positive feed forward loop, thus potentiating 5-LO/LTB4/BLT signalling [176] (Figure 5). Furthermore, a role of 5-LO in the modulation of lipid metabolism, and increasing lipogenesis by adipocytes and thus the availability of FFAs for pro-inflammatory eicosanoid synthesis has also been highlighted [172].

Recent studies have shown that obesity results in a shift in the immune profile of the adipose tissue from M2 macrophages and TH2 and regulatory T-cells towards pro-inflammatory TH1, cytotoxic CD8+ T-cells and M1 macrophages [177]. This chronic low-grade inflammation of adipose tissue has been shown to potentiate associated obesity-driven pathologies [167,178]. Pharmacological inhibition or genetic deletion of 5-LO enzyme, FLAP or the 5-LO metabolite receptors in adipocytes or adipose tissue has been shown to cause an anti-inflammatory phenotype, with reduced chemotactic potential, lower adipose tissue macrophage (ATM) infiltration, decreased M1:M2 phenotype in the stromal vascular fraction and decreased FFA release [170,172,179,180,181].

Given the similar histology of obese adipose tissue inflammation in both metabolic disease and cancer [165], the evidenced role of adipose-tissue 5-LO signalling in metabolic disease [170,172] and the role of 5-LO in components of the TME [37], it is possible, although not yet investigated, that adipose-tissue derived 5-LO activity plays a role in the TME. Leukotrienes and HETEs produced by the adipocytes or adipose tissue stromal vascular fraction may act locally within the fat or they have the potential be released systemically to stimulate cancer cells and stromal cells bearing leukotriene receptors. Additionally 5-LO induced lipogenesis and subsequently released FFAs in the TME have the potential to be utilised by more distal cellular sources with AA metabolising activity, such as cancer cells and associated stromal cells [182]. Obesity associated adipokines such as leptin, TNF-α and IL-6 have already been shown to be involved in the development and progression of cancer [183,184]. Similar investigations into the potential role of obesity associated 5-LO signalling specifically in cancer are also warranted. Figure 5 summarises the evidenced effect of upregulated 5-LO signalling in obese adipose tissue and the effect of blocking this pathway in adipose tissue.

## 7. 5-Lipoxygenase and Cancer-Associated Fibroblasts

Interactions of tumour cells with stromal cells of the microenvironment are crucial to cancer progression, i.e., stimulating blood vessel growth and development and activation of pro-inflammatory cells and immune suppressor cells. Additionally another subset of stromal cells, fibroblasts, have been shown to play an important role in the growth and spread of a tumour [182]. Fibroblasts tend to be increased in tumour-associated stroma compared to normal stroma. Fibroblasts in the vicinity of the tumour are thought to obtain an activated phenotype and they are termed cancer associated fibroblasts (CAFs), identified by the expression of alpha smooth muscle actin (α-SMA) [185]. These CAFs are thought to form the majority of stromal fibroblasts in tumours. CAFs interact with cancer cells and other stromal components such as endothelial cells and immune cells through the production of growth factors, including VEGF and chemokines. In addition CAFs regulate extracellular matrix (ECM) composition and turnover by the deposition of ECM constituents such as collagen types I and III or secretion of ECM-degrading proteases such as matrix metalloproteinases [185].

Similar to leukocytes, LTB4 has been shown to induce chemotaxis of normal fibroblasts in culture [186]. Fibroblasts express LT receptors [187,188] and similar to endothelial cells, fibroblasts do not express 5-LO but have been shown to express LTA4 hydrolase. Thus, they are capable of transcellular leukotriene synthesis when provided with an external source of the intermediate metabolite LTA4, such as infiltrating leukocyte LTA4 [189] (Figure 2). Leukotrienes have been shown to be involved in fibroblast-associated non-cancerous pathologies such as hepatic and pulmonary fibrosis [190,191]. There is currently no research in the area of leukotriene synthesis and CAFs, however, Medina et al. demonstrated that fibroblasts which had been transformed by simian virus 40 (SV-40) had significantly increased LTA4 hydrolase activity and produced more LTB4 compared to normal fibroblasts [192]. This raises the possibility that altered fibroblasts in the TME of a tumour with 5-LO activity could be involved in inducing inflammation and metastasis through an upregulation of LTB4.

Taylor et al. have provided evidence of cross-talk between cancer cells and fibroblasts that involves, at least partially, 5-LO signalling [192] They demonstrated that breast cancer cells released a glycoprotein called EMMPRIN that can induce pro-MMP2 release by fibroblasts. Pharmacological inhibition indicated that MMP2 release occurred through the activation of 5-LO in the breast cancer cells while addition of 5-HETE was shown to potentiate the effects of the breast cancer cell conditioned media [192]. Therefore, targeting MMPs and 5-LO may disturb important interactions between a growing tumour and the ECM and thus provide a potential therapeutic route in the treatment of metastatic disease. The role of 5-LO-LT signalling pathway in fibroblasts, in the context of cancer is a relatively under researched area. Considering the rising interest in CAFs and the strong association of eicosanoid signalling pathways with tumour development and progression, it is an area that warrants investigation.

## 8. Conclusions

A strong link between inflammation and cancer has been established for many years and while our knowledge is constantly improving, many aspects of the mechanistic link between cancer and inflammation are not well understood. Considering their important role in inflammation, 5-LO metabolites may have a linking role between these two processes. Most 5-LO inhibitory studies have focused mainly on targeting the tumour promoting effects of this pathway in cancer cells. However, as evidenced in this review, there is large potential for cross-talk of cancer cells with stromal cells including immune cells and connective tissue components such as endothelial cells, adipocytes and fibroblasts and indeed for the crosstalk of stromal cells with each other through the 5-LO pathway, as summarised in Figure 2.

Given the increased awareness of the important role of the TME in tumour growth and development, the 5-LO pathway provides an interesting route of investigation for a TME-focused therapeutic strategy to prevent or halt tumorigenesis. 5-LO pro-inflammatory signalling through immune cells has the potential to be both pro- or anti-tumorigenic, therefore the identification of what cells types and pathways shift this balance in favour of one direction over the other in a particular cancer is important. Further investigation is also needed to understand at what stage in tumour initiation 5-LO cross-talk signalling between a cancer cell and the TME stimulates cancer development. Finally, the role of adipose tissue derived 5-LO metabolites in driving obesity-associated inflammatory disorders and potentially obesity-driven cancer is an important avenue of research.

## Abbreviations

12-LO	12-Lipoxygenase
15-LO	15-Lipoxygenase
5-LO	5-Lipoxygenase
AA	Arachidonic acid
BAL	Bronchial alveolar lavages
COX	Cyclooxygenase
ECM	Extracellular matrix
FFA	Free fatty acids
FLAP	5-Lipoxygenase associated protein
HETE	Hydroxyeicosatetraenoic acid
LT	Leukotriene
MDSC	Myeloid-derived suppressor cells
PMNL	Polymorphonuclear leukocytes
TAM	Tumour associated macrophages
TME	Tumour microenvironment
